# Colony-Stimulating Factors on Mobilizing CD34^+^ Cells and Improving Neurological Functions in Patients With Stroke: A Meta-Analysis and a Systematic Review

**DOI:** 10.3389/fphar.2021.704509

**Published:** 2021-07-22

**Authors:** Xiuqi Chen, Wenbo Sun, Ping Zhong, Danhong Wu

**Affiliations:** ^1^ Department of Neurology, Shanghai Fifth People’s Hospital, Fudan University, Shanghai, China; ^2^ Department of Neurology, Shidong Hospital Affiliated to University of Shanghai for Science and Technology, Shanghai, China

**Keywords:** stroke, colony-stimulating factor, meta-analysis, therapy, neurological diseases

## Abstract

**Background and Purpose:** CSF therapy is considered a promising therapeutic approach for stroke. We performed a meta-analysis to explore the safety and efficacy of CSF in published clinical stroke studies.

**Methods:** We searched articles online and manually. Two reviewers selected studies independently, selecting data based on study quality, characteristics of intervention (administration time, observation time, type, dose, and injection approach of CSF), and the baseline characteristics of patients (age, sex, hypertension, diabetes, smoker, and lipids) were extracted. Main prognosis outcomes were measured as all-cause death in severe adverse events (SAE) and recurrent stroke in SAE. Secondary outcomes were measured as CD34+ cell counts in periphery blood at day 5, National Institutes of Health Stroke Scale (NIHSS), and Barthel index (BI), Side effects of CSF were taken as the indicator of safety. STATA13 software was used to perform the meta-analysis.Keywords: Stroke, Colony-stimulating factor, Meta-analysis, therapy, Neurological Diseases

**Results:** This meta-analysis involved 485 patients from eight studies. Among them, 475 patients from seven studies were gauged SAE (all-cause death), 393 patients from six studies were checked SAE (recurrent stroke); 137 patients from three studies underwent CD34^+^ measurement, 389 patients from six studies were tested NIHSS and 307 patients from five studies accessed BI. Compared with the control group, both all-causes death (RR= 1.73, 95%CI= (0.61, 4.92), P=0.735, I^2^=0.0%) and recurrent stroke (RR= 0.43, 95%CI= (0.14, 1.32), P=0.214, I^2^=33.1%) present no statistical differences, indicating that the application of CSF does not statistically alter the prognosis of patients with stroke. The application of CSF effectively enhanced CD34+ cell counts in periphery blood at day 5 (SMD= 1.23, 95%CI= (0.54, 1.92), P=0.04, I^2^=69.0%) but did not statistically impact NIHSS (SMD= −0.40, 95%CI= (−0.93, 0.13), P ≤ 0.001, I^2^=79.7%) or BI (SMD= 0.04, 95%CI= (−0.38, 0.46), P=0.068, I^2^=54.3%).

**Conclusion:** Our study consolidates the security of CSF administration for its exerting no effect on detrimental outcomes. It has proven to be effective in elevating CD34+ cell counts in periphery blood at day 5, indicating CSF may participate in stroke recovery, but its efficacy in stroke recovery remains detected.

**Systematic Review Registration:** PROSPERO, CRD42020166216.

## Introduction

Stroke causes great burdens to society worldwide, especially in low-income and middle-income countries (Krishnamurthi et al., 2013). In China, stroke is the leading cause of death and disability-adjusted life-years (DALYs) (Zhou et al., 2019). Although emergency treatments like thrombolysis and mechanical embolectomy can save the lives of patients and promote neurological recovery, the use is limited by the strict administration conditions and severe side effects of these treatments (Bravata, 2005; Adams et al., 2007; Thomalla et al., 2007; Lambrinos et al., 2016). Thus, new therapeutic approaches are necessary for better recovery.

CSF is a group of glycoprotein, including granulocyte colony-stimulating factor (G-CSF), erythropoietin (EPO), macrophage colony-stimulating factor (M-CSF), granulocyte-macrophage colony-stimulating factor (GM-CSF), and interleukin-3 (IL-3), etc. (Wakefield et al., 1990) They were first discovered in bone marrow cells that had been cultured agar (Ichikawa et al., 1966; Landau and Sachs, 1971) and since then, increasing works have been down on CSFs. They have been demonstrated to enhance the proliferation and differentiation of stem cells, stimulate angiogenesis and neurogenesis as well as produce antiapoptotic function (Jung et al., 2006; Komine-Kobayashi et al., 2006; Greenbaum and Link, 2011; Kimáková et al., 2017), serving as promising candidates after stroke. Schäbitz and his colleagues found that G-CSF directly protects cells from glutamate-induced excitotoxicity *in vitro* and diminishes infarct volume after experimental stroke *in vivo* by targeting G-CSF receptors on neuron membrane (Schäbitz et al., 2003). Another study also reported the protective function of G-CSF in increasing survival rate and decreasing infarct volume, but they assumed that this function may due to the mobilization function of G-CSF on bone marrow stem cells. The mobilized bone marrow stem cells may differentiate into nervous and endothelial cells and repair the injured zone (Six et al., 2003). This assumption has been corroborated in later studies. One study verified that G-CSF mobilized peripheral blood progenitor cells leading to a significant reduction of stroke-induced hyperactivity (Willing et al., 2003), and compared with stem cells not pre-treated, pre-treated stem cells manifest stronger proliferation and improve motor function quicker (Hokari et al., 2009). Another study found that with G-CSF injection, more stem cells honed in on the subventricular area of injured brain tissue (Shyu et al., 2004). Not only bone marrow (BM) derived stem cells are mobilized by CSF. Administration of G-CSF and stem cell factor (SCF) in the subacute phase after focal cerebral ischemia contributes to neuronal generation from both BM-derived cells and intrinsic neural stem/progenitor cells (Kawada et al., 2006). Though CSF mobilized stem cells may exert protective function in stroke, we lack direct proof supporting the fact that the protections are due to stem cells differentiating into neurons and repairing the injured brain. Toth et al. found that most cells entering the brain maintained bone marrow identity and did not transdifferentiate into neural cells, but G-CSF combined with SCF administration and resulted in a 1.5-fold increase in vessel formation, thus the neuronal survival was attributed to the consequence of CSF-induced angiogenesis (Toth et al., 2008). Endogenous CSFs are also essential for brain recovery after stroke (Sevimli et al., 2009) that G-CSF-deficient mice manifested larger infarct volume, while G-CSF substitution rescues these detrimental effects (Strecker et al., 2010). Though it seems that CSFs are hopeful for stroke recovery, inconsistent outcomes from clinical trials challenge whether CSFs are tolerable and efficient for improving human neurological function after stroke. This meta-analysis aims to obtain the overall expression of the function of CSF on stroke and for better guiding research in the future. In this study, we include randomized controlled trials (RCT) targeting adult patients with acute, subacute, or chronic stroke and analyze if CSF administration makes differences in SAE, stem cell mobilization (CD34 ^+^ cell counts), and neurological prognosis (NIHSS and BI). Considering the heterogeneity that exists in both interventions and the baseline characteristic data of patients, we investigated the combined effects of the time of injection (administrationNo), the time of observing outcome (observationNo), the dose of CSF (doseNo), type of CSF (interventionNo), and baseline characteristics and for this purpose, we used the meta-regression technique (Thompson and Higgins, 2002).

## Materials and Methods

We did this meta-analysis in accordance with the Preferred Reporting Items for Systematic Reviews and Meta-Analyses (PRISMA) reporting guidelines (Moher et al., 2009).

### Retrieving the Literature

We searched the PUBMED, EMBASE, and Cochrane library databases. The PUBMED strategy was depicted as follows: (((((((“Stroke” [Mesh]) OR (cerebrovascular accident*[Title/Abstract])) OR (cerebrovascular apoplexy [Title/Abstract])) OR (accident*, cerebrovascular [Title/Abstract])) OR (brain vascular accident*[Title/Abstract])) OR (stroke [Title/Abstract])) AND (((((((((((((((“Colony-Stimulating Factors” [Mesh]) OR (CSF [Title/Abstract])) OR (Granulocyte colony-stimulating factor [Title/Abstract])) OR (G-CSF [Title/Abstract])) OR (erythropoietin [Title/Abstract])) OR (EPO [Title/Abstract])) OR (macrophage colony-stimulating factor [Title/Abstract])) OR (M-CSF [Title/Abstract])) OR (granulocyte-macrophage colony-stimulating factor [Title/Abstract])) OR (GM-CSF [Title/Abstract])) OR (interleukin-3 [Title/Abstract])) OR (IL-3 [Title/Abstract])) OR (Thrombopoietin [Title/Abstract])) OR (TPO [Title/Abstract])) OR (Colony-stimulating factor*[Title/Abstract]))) AND ((((((randomized controlled trial) OR (randomized)) OR (placebo)) OR (clinical trials, randomized)) OR (RCT)) OR (trials, randomized controlled)). We included articles in English. The bibliographies were cross checked respectively by two investigators. A third investigator resolved issues when divisions arose.

### Selection of Studies and Data Extraction

Inclusion criteria: 1) the design of the included study was an RCT on stroke with adult population; 2) the intervention involves the administration of one kind of CSF in stroke; 3) the research reported one or more outcome measures including NIHSS, BI scores, SAE, and laboratory values for CD34^+^; 4) the subjects were diagnosed with stroke by clinical manifestation, laboratory tests and magnetic resonance imaging (MRI) or computerized tomography (CT), despite ischemic or hemorrhagic, acute, subacute or chronic.

SAE (all-cause death) and SAE (recurrent stroke) were considered as the main outcome. CD34^+^ cell counts in periphery blood at day 5, NIHSS (an inventory reflecting the severity of the stroke, with higher scores indicating more severe stroke), and BI scores (an inventory reflecting the ability to live independently, with lower scores indicating more severe functional disorders) as efficacy outcomes were also recorded.

### Quality Assessment

The methodological quality of included RCTs was assessed critically by two reviewers according to Cochrane Handbook 5.1. Each study was rigorously appraised in accordance with the following seven scales: 1) random sequence generation; 2) allocation concealment; 3) blinding of participants and personnel; 4) blinding of outcome assessment; 5) incomplete outcome data; 6) selective reporting; 7) other bias. The quality of the evidence in individual studies was graded according to the GRADE workgroup recommendation by two researchers (Atkins et al., 2004).

### Statistical Analysis

We calculated the standardized mean difference (SMD) and the relative ratio (RR) and their respective 95% confidence interval (CI) as measures of the relationship between CSF and outcomes. RR was calculated for dichotomous variables and SMD for continuous variables. I^2^fn2 statistic was used to determine heterogeneity. We performed meta-analysis using a fixed effects model to achieve the pooled RR and SMD within the included studies, a random-effects model was used when statistically significant heterogeneity was existent (I^2^>50%, *p* < 0.05) or I^2^>50% but proves no statistical significance (*p* > 0.05). If the heterogeneity was still not eliminated, the sensitivity analysis and meta-regression would be applied. All analyses were conducted by Stata 13.0. A *p* value of less than 0.05 was considered statistically significant.

## Results

### Study Inclusion and Study Characteristics

**FIGURE 1:**
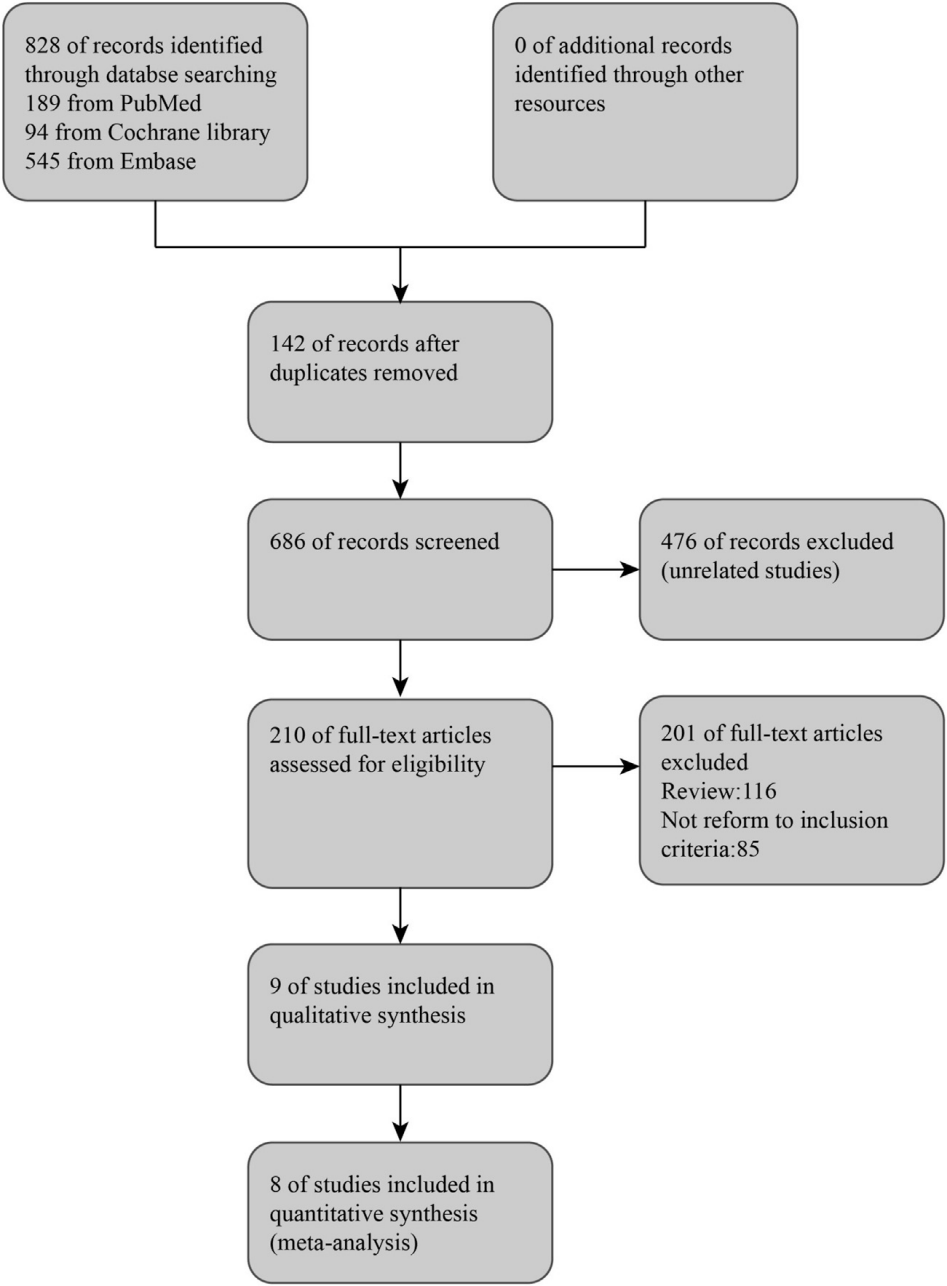
Flow chart.

From online searches, nine abstracts that met the inclusion criteria were chosen. One research reported that their data did not obey normal distribution, and the sample size was quite small (three experimental groups to three matched groups). It is not appropriate to transform the interquartile range into mean and standard deviation. Therefore, this article was excluded (Alasheev et al., 2011). Manual searching did not identify any further eligible data. (see Figure 1). Thus, the meta-analysis is based on the data from eight articles (Table1). Outcomes were assessed in a total of 475 patients for SAE (all-cause death) and 393 patients for SAE (recurrent stroke), 137 patients for CD34^+^ measurements, 389 patients for NIHSS, and 307 patients for Barthel Index. A combination of administration time, observing time, CSF dose, CSF type, and baseline characteristics (age, hypertension, diabetes, abnormal plasma lipid condition, sex, smoker) in the studies were presented separately for comparisons of NIHSS and Barthel Index (see Supplementary Figures S1, S2).

**TABLE1:** Clinical studies of CSF in stroke.

First author	Trial design	Intervention	Outcomes	Stroke type	Age, yr (mean)	Male^a^	Hypertension^a^	Diabetes^a^	Smoker^a^	Lipids^a^
Year of publication										
Shyu et al. (2006)	Randomized, blinded controlled trial	G-CSF, subcutaneous, 15 μg/kg/day for 5 days	BC	Acute ischemic stroke	65.4	60.0	100.0	60.0	20.0	40.0
Sprigg et al. (2006)	Randomized, blinded controlled trial	G-CSF, subcutaneous	AD	Subacute ischemic stroke	75.3	50.0	66.7	-	-	27.8
		1/3/10 μg/kg, 1/5/day for 5 days								
Prasad et al. (2011)	Randomized, open-label, controlled trial	G-CSF, subcutaneous	BCD	Acute ischemic stroke	46.4	-	20.0	0	20.0	20.0
		10 μg/kg,1/day, 5 days								
Yip et al. (2011)	Randomized, blinded controlled trial	EPO, subcutaneous	BCD	Acute ischemic stroke	65.4	65.9	68.9	34.7	59.9	-
		5000IU/per time								
Timothy et al., 2012	Randomized, blinded, controlled trial	G-CSF, subcutaneous	ABCD	Subacute ischemic/hemorrhagic stroke	71.5	32.0	31.0	6.0	-	27.0
		10 μg/kg, 1/day, 5 days								
Sprigg et al. (2016)	Randomized, blinded controlled trial	G-CSF, subcutaneous	AD	Chronic stroke	66.2	60.0	78.0	21.0	-	-
		10 μg/kg, 1/day, 5 days								
Zhou et al. (2019)	Randomized, open-label, controlled trial	rhG-CSF, subcutaneous	BD	Acute ischemic stroke	63.8	68.3	78.0	35.3	-	-
		300 µg,2/day, 5 days								
Gorthi 2018	Randomized, open-label, controlled trial	G-CSF,subcutaneous	BCD	Acute ischemic stroke	55.6	61.7	53.3	36.7	38.3	-
		15 μg/kg/day, 5 days								

A: CD34^+^ cell counts in periphery blood at day 5.

B: NIHSS.

C: BI.

D: SAE.

a Figures represent percentage (%).

-Data not available.

### Study Quality

**FIGURE 2:**
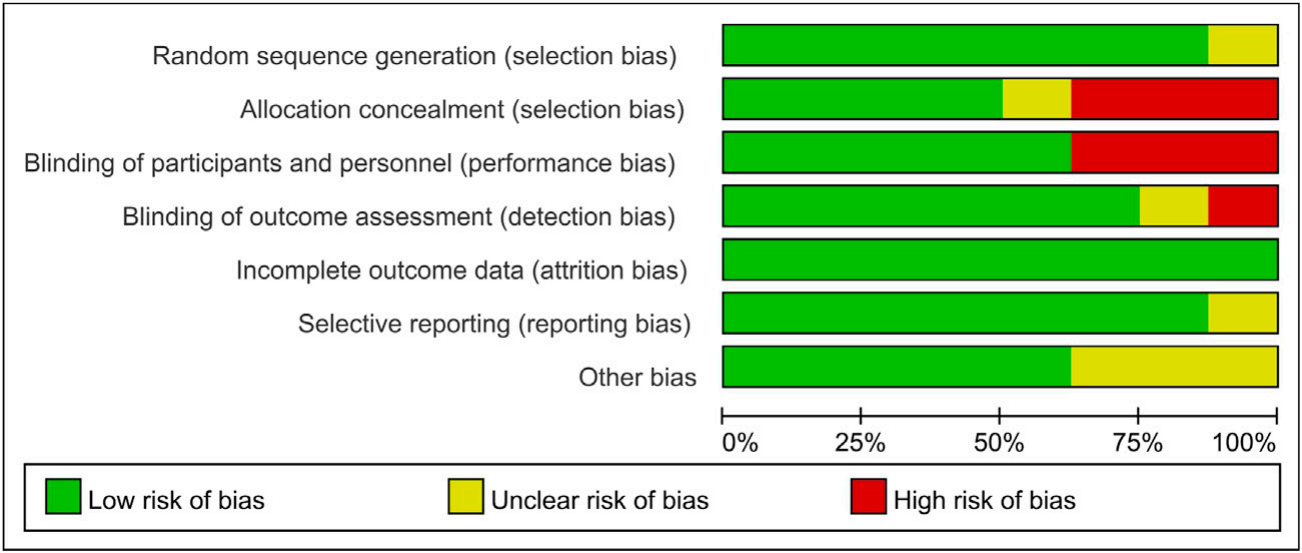
Risk of bias graph.

**FIGURE 3:**
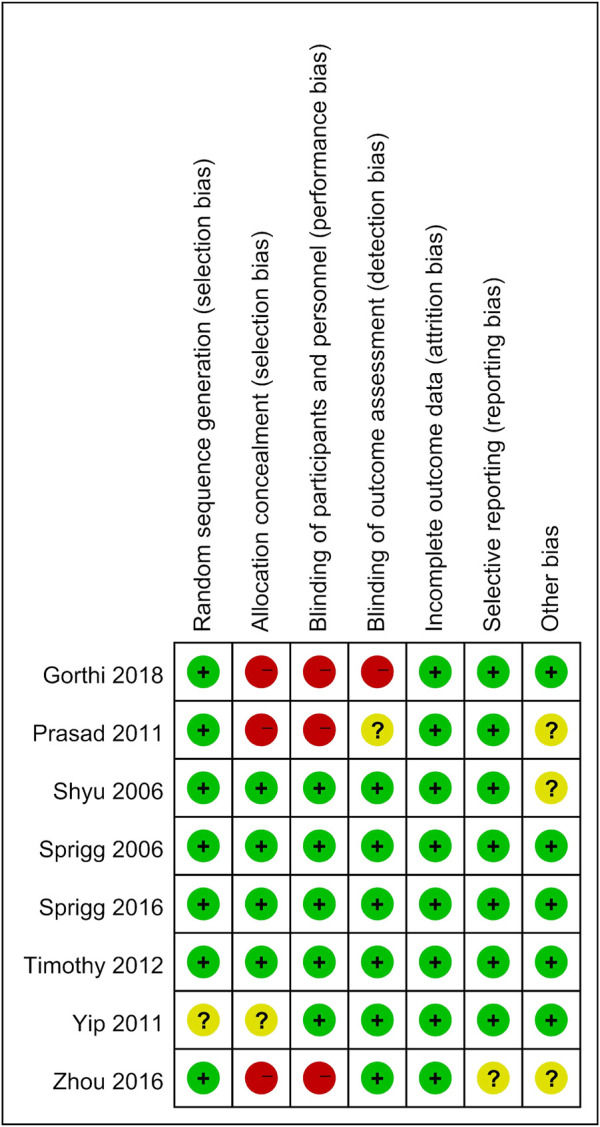
Risk of bias summary.

**FIGURE 4:**
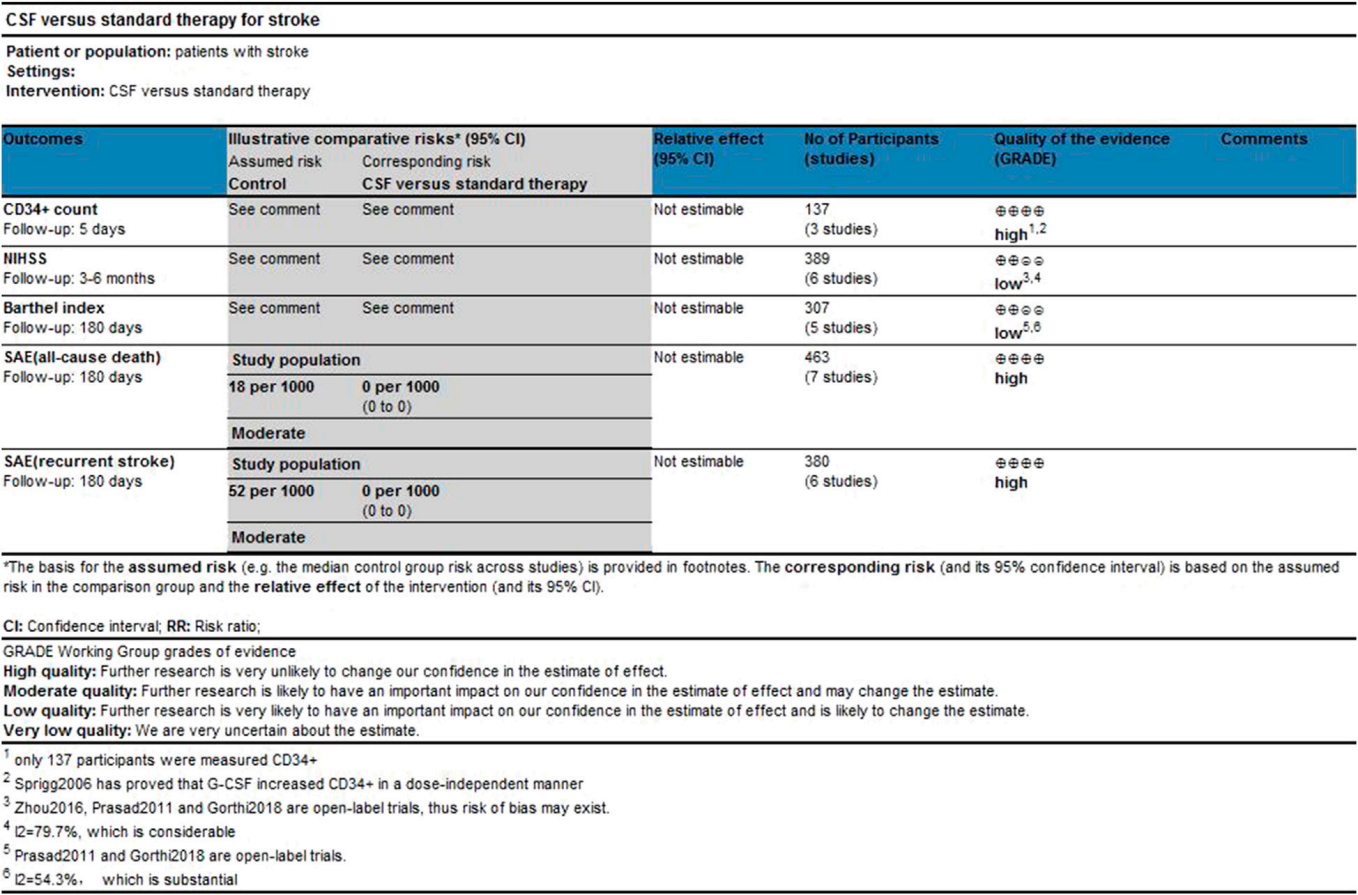
Gradepro assessment.

Two researchers assessed the quality of the included studies independently by using ReVman (see Figures 2, 3). Three out of the eight articles are open-labeled, thus presenting high risk in terms of “allocation concealment” and “blinding of participants and personnel”. In these three open-labeled studies, Zhou depicted that the outcomes were all analyzed by researchers blinded to patient groupings. Prasad (Prasad et al., 2011) did not refer to the blinding of outcome assessment. Gorthi showed that patients groupings were transparent to all participants. Thus, in the part of “Blinding of outcome assessment”, Zhou was ranked as “low risk”, Prasad was graded by “unclear risk” while Gorthi presented “high risk”. All studies presented low risks in terms of “attrition bias” and most studies present low or unclear risks in “selection bias”, “reporting bias” and “other bias”. The quality of the evidence in individual studies was graded according to the GRADE workgroup recommendation by two researchers (See Figure 4).

### Efficacy, Safety and Meta-Regression Analysis

#### Efficacy

**FIGURE 5:**
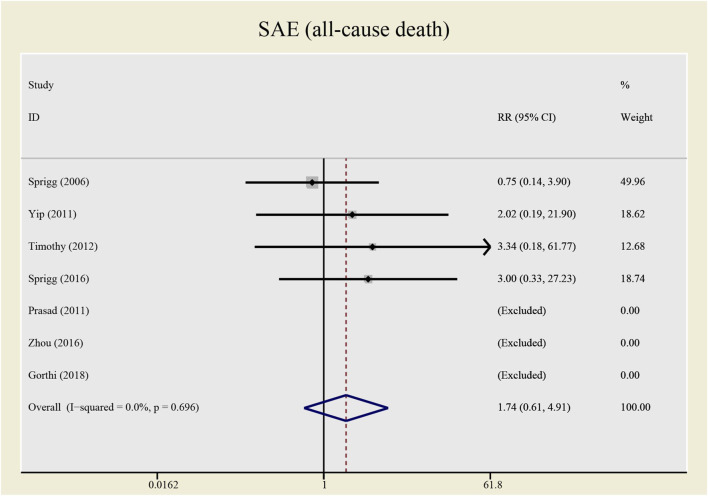
Forrest plot for SAE (all-cause death).

**FIGURE 6:**
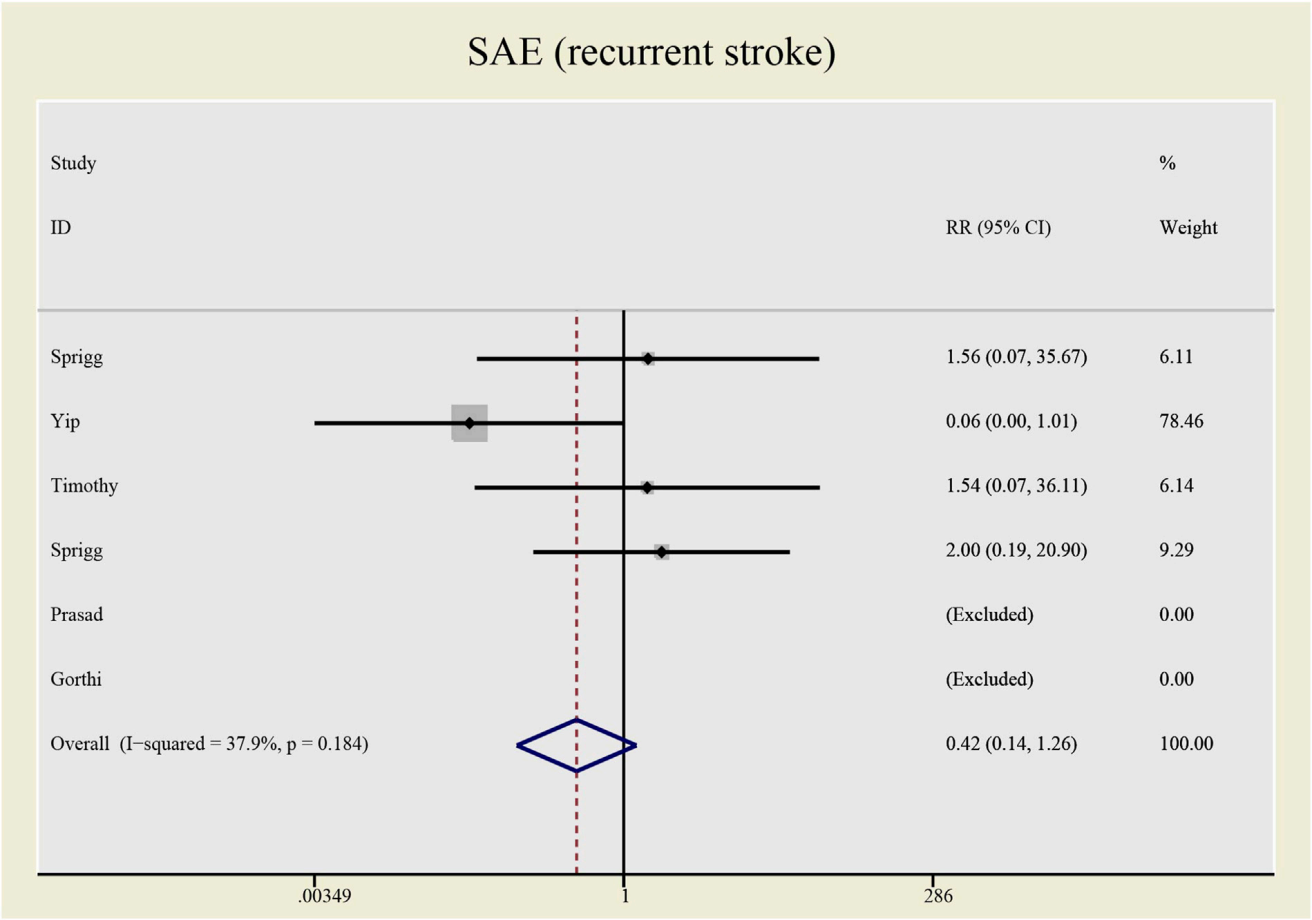
Forrest plot for SAE (recurrent stroke).

No significant differences were found in the heterogeneity test in all-causes deaths (I^2^ = 0.0%) and recurrent stroke (I^2^ = 33.1%). Both of the I^2^ are lower than 50%, thus a fixed effect model was employed to combine effect size. Even though the CSF administration seems to have no effects on prognosis (see Figures 5, 6) that all-causes death (RR = 1.73, 95%CI= (0.61, 4.92), *p* = 0.735, I^2^ = 0.0%) and recurrent stroke (RR = 0.43, 95%CI= (0.14, 1.32), *p* = 0.214, I^2^ = 33.1%) present no statistical differences, SAE (all-causes death) was prone to increase while SAE (recurrent stroke) tended to decrease.

**FIGURE 7:**
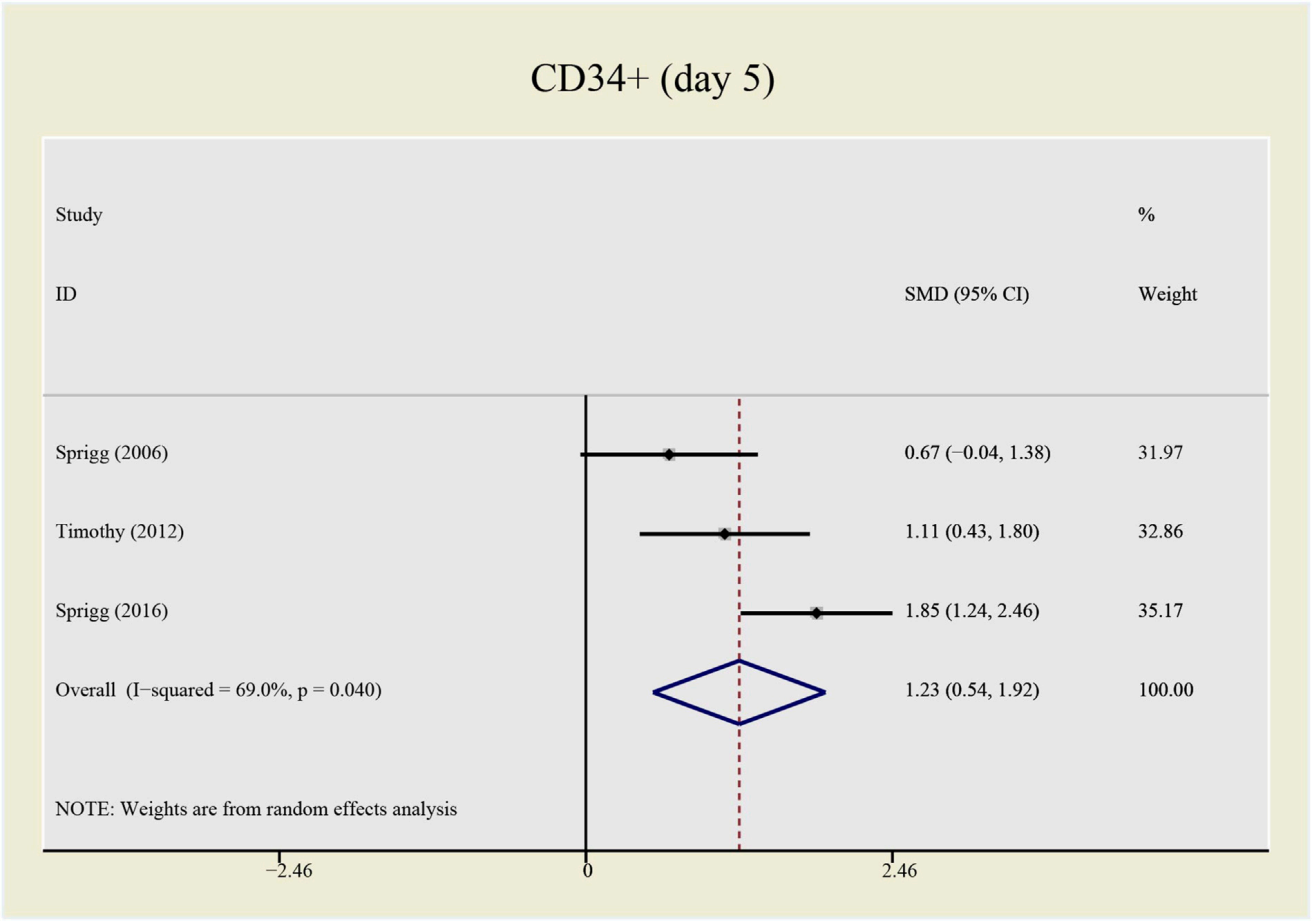
Forrest plot for CD34^+^ at day 5.

**FIGURE 8:**
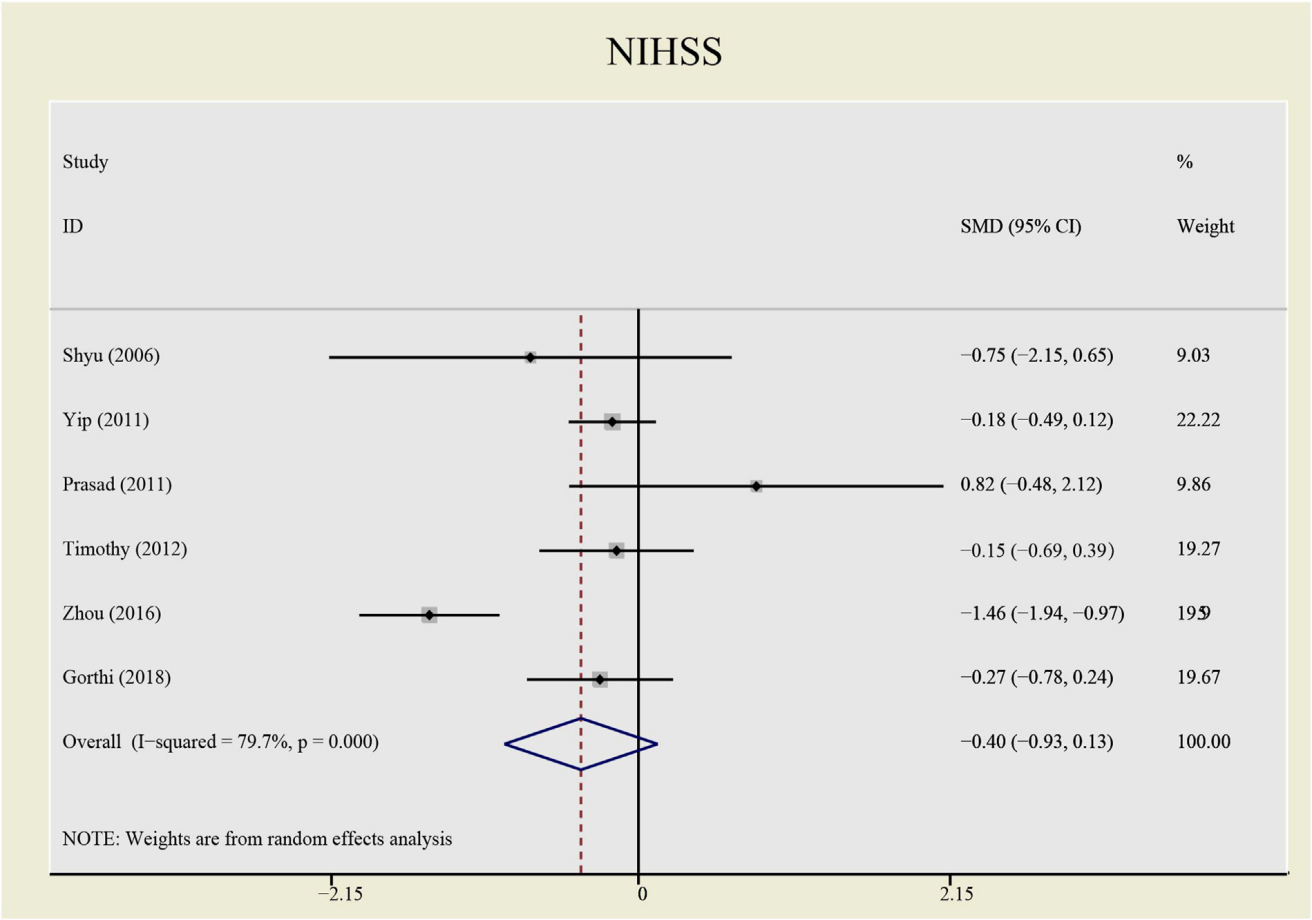
Forrest plot for NIHSS.

**FIGURE 9:**
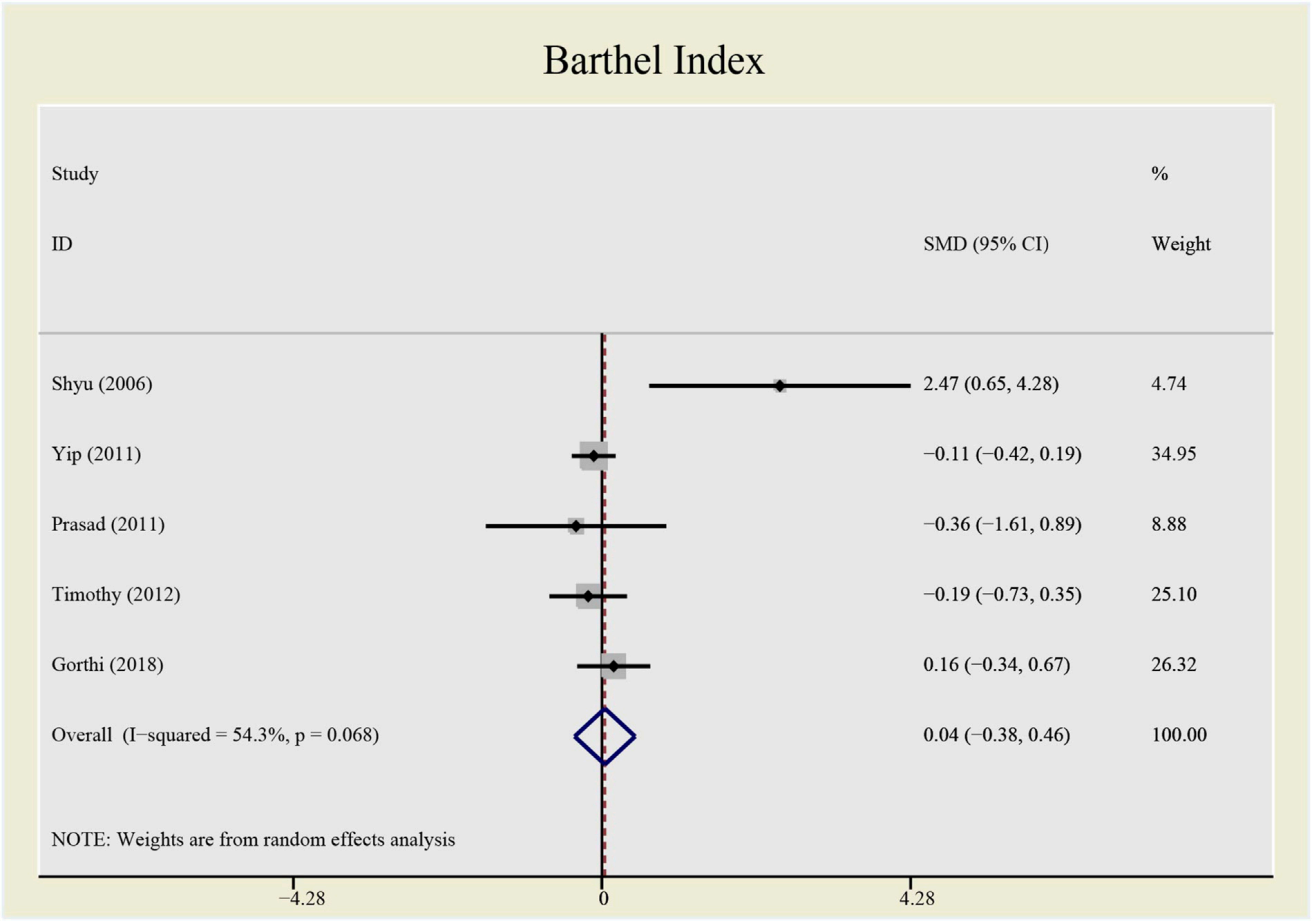
Forrest plot for Barthel Index.

The heterogeneity test presents significant differences in CD34^+^ (I^2^ = 69%), NIHSS (I^2^ = 79.7%) and BI (I^2^ = 54.3%), a random effects model was chosen here. CSF administration elevates CD34 ^+^ level at day 5 (SMD = 1.23, 95%CI= (0.54, 1.92), *p* = 0.04, I^2^ = 69.0%) significantly, but does not impact NIHSS (SMD = -0.40, 95%CI= (-0.93, 0.13), *p* ≤ 0.001, I^2^ = 79.7%) nor BI (SMD = 0.04, 95%CI= (-0.38, 0.46), *p* = 0.068, I^2^ = 54.3%) (see Figures 7–9). It indicates that even if the CSF application mobilizes CD34^+^, the neurological tests do not show significant changes.

#### Safety

Shyu (Shyu et al., 2006) reported that three participants in the experimental group claimed headache, bone pain, and transient liver function abnormality. Prasad (Prasad et al., 2011) reported one case of bone pain and one case of deep venous thrombosis in the experimental group. Sprigg (Sprigg et al., 2016) reported that in the experimental group, six people claimed bone pain, three people outlined that they had headache, and no one had been infected while in the controlled group. Three people claimed bone pain, four persons claimed headache, and two got infected. Zhou et al., 2019 had reported that gastrointestinal reactions had been found in 19 people, and bone pain was found in 15 people, fever was found in 12 people and deep venous thrombosis was found in one person in the experimental group, while no other side effects had been found in the control group. Overall, they found the application of CSF to be safe.

#### Meta-Regression Analysis

We conducted meta-regression to find out the cause of high heterogeneities. However, neither administration factors (the time of injection, the time of observing outcome, dose of CSF, and type of CSF) nor baseline characteristics of the patients altered the effects of CSF treatment (see Supplementary Figures S1, S2) on NIHSS or BI.

**FIGURE 10:**
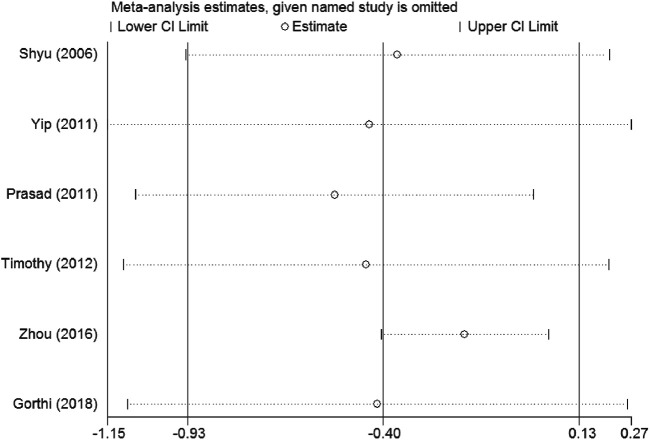
Sensitivity analysis for NIHSS. CI, confidence interval.

**FIGURE 11:**
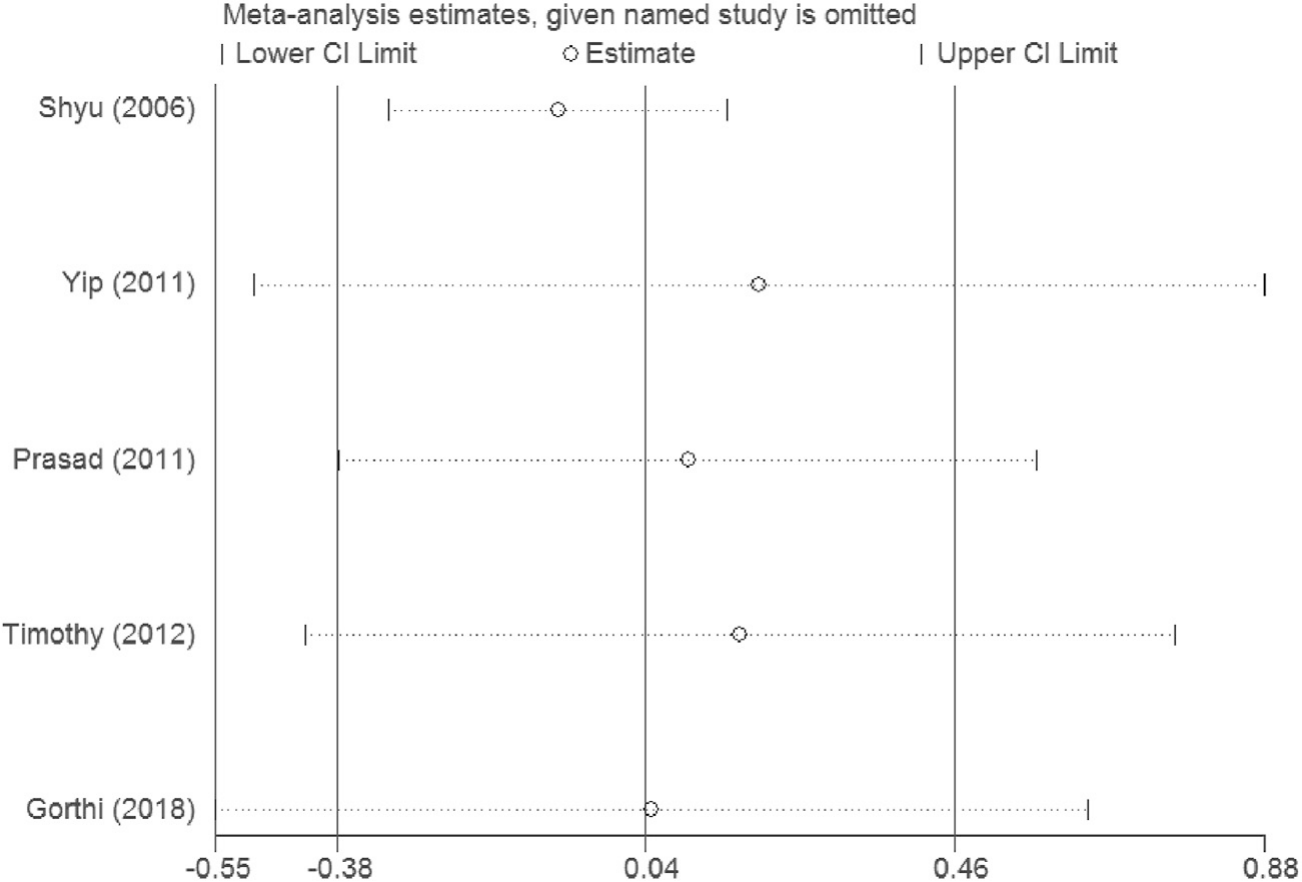
Sensitivity analysis for BI. CI, confidence interval.

Sensitivity analysis demonstrates that the overall result is not impacted by dismissing research in each turn, and our outcomes are reliable (see Figures 10, 11).

### Publication Bias

**FIGURE 12:**
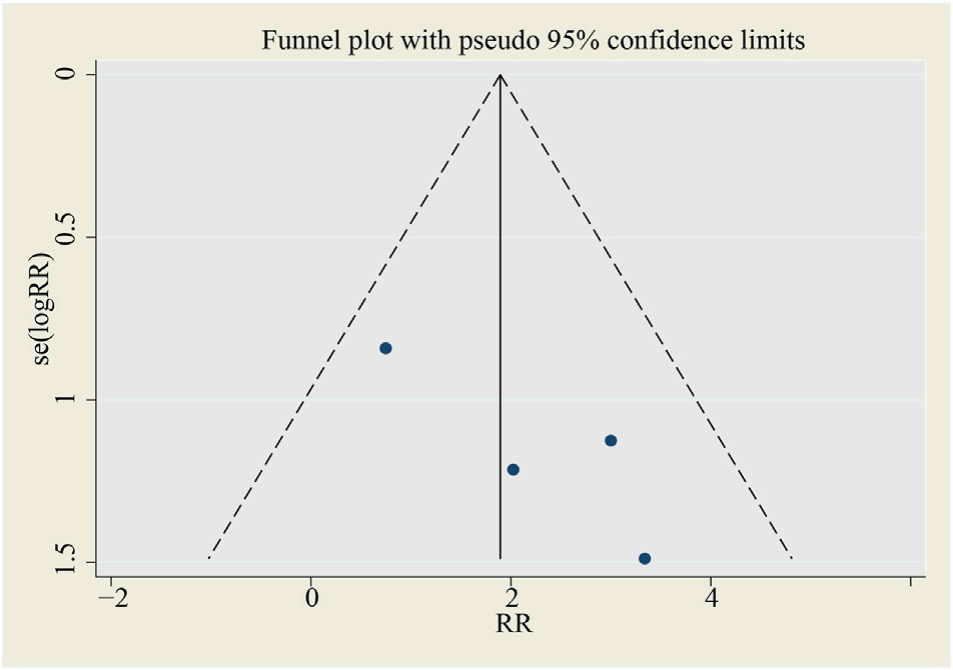
Funnel plot for SAE (all‐cause death). SMD, standardized mean difference, SE, standard error.

No publication bias is found in the funnel plot (see Figure 12).

## Discussion

### Safety and Efficacy of Colony-Stimulating Factors on Stroke

This meta-analysis includes the newest research and demonstrates the safety of CSF administration on stroke recovery. Interestingly, though no statistical significance has been found, CSFs tend to increase the onset of SAE (all-cause death) and decrease SAE (recurrent stroke), implying that CSF treatment is a promising therapeutic approach for patients with stroke. Though there is a trend that CSF may be of benefit for neurological improvement (NIHSS and BI), no significant outcomes have been drawn. To confirm its safety and effectiveness, a larger scale of RCTs will be needed in the future.

Most preclinical studies corroborate the efficacy of CSFs for stroke recovery. CSFs achieved a neuroprotective function by directly binding CSF receptor (Schäbitz et al., 2003; Villa et al., 2003) or by indirectly mobilizing BM-derived stem cells (Six et al., 2003; Willing et al., 2003) and intrinsic neural stem/progenitor cells (Kawada et al., 2006) to the subventricular zone (SVZ). This protection remains when administered in the acute, subacute phase (Kawada et al., 2006) and chronic phase (Schneider et al., 2006; Zhao et al., 2007; Piao et al., 2009; Larpthaveesarp et al., 2016) of stroke. G-CSF is also reported to have a survival-enhancing capacity and a beneficial effect on the functional outcome for aged rats (Popa-Wagner et al., 2010). The beneficial effects of CSFs are via anti-apoptosis (Kretz et al., 2005; Schneider et al., 2005; Li et al., 2007; Mengozzi et al., 2012; Souvenir et al., 2014; Komnig et al., 2018), anti-inflammation (Villa et al., 2003; Dietel et al., 2012; Mengozzi et al., 2012; Yuen et al., 2017) angiogenesis (Li et al., 2007; Liu et al., 2010), neurogenesis (Kretz et al., 2005; Tsai et al., 2006; Gonzalez et al., 2013), oligodendrogenesis (Zhang et al., 2010; Gonzalez et al., 2013; Wang et al., 2017), and the protection for synaptic functions and axonal myelination (Schneider et al., 2005; Liu et al., 2010; Zhang et al., 2010; Mengozzi et al., 2012). It is also reported that CSFs can protect the brain after stroke by ameliorating astrocyte swelling and maintaining the brain liquid equilibrium (Gunnarson et al., 2009) or remodeling the neuron-vascular network (Cui et al., 2015). Besides direct interventions, some research corroborates that CSFs are effective in extending the therapeutic time window of t-PA and thereby indirectly enhancing the possibility of stroke recovery (dela Peña et al., 2015; Dela Peña et al., 2018). Moreover, some analogues of EPO are studied as well (Villa et al., 2007; Gan et al., 2012). They are proven to produce fewer side effects but generate similar protective effects, and are currently regarded as a brand-new and hopeful therapy for stroke (Gan et al., 2012).

Though CSFs seemed promising, not all preclinical research draws positive conclusions about CSFs on stroke. One article reported that EPO did not protect brain tissue after stroke, and if the plasma level of EPO is too high, it is detrimental for elevating hematocrit and increases accompanying blood viscosity (Wiessner et al., 2001). Another study demonstrated that G-CSF might lead to negative outcomes in stroke by exaggerating inflammatory responses (Taguchi et al., 2007). The fact that CSFs are detrimental under certain circumstances partly accounts for the tendency that CSF increases the onset of SAE and indicates that all factors that influence CSFs plasma level (including the dose and application approach) may affect the outcome, and this is the reason we select the dose of CSF and other details of interventions to detect the origins of heterogeneity. Age and comorbidities like diabetes have also been reported to influence the function of G-CSF on stroke in preclinical studies (Lan et al., 2008; Popa-Wagner et al., 2010; Buga et al., 2013). Thus, we explore whether these factors will impact the function of CSFs on stroke in this meta-analysis (see Supplementary Figures S1, S2). However, none of them have been proven to make a significant difference. This may be in part due to the limited number of enrolled trials and missing data, partly owing to some undetected factors. For example, though several studies have corroborated that CSFs rescue ischemic tissue through anti-inflammation function (Villa et al., 2003; Dietel et al., 2012; Mengozzi et al., 2012; Yuen et al., 2017), another study claims that G-CSF will induce an exaggerated inflammatory response, thus leading to cortical atrophy and impaired behavioral function (Taguchi et al., 2007). Some conditions causing the transformation of the role of CSFs in inflammation do exist, and some other stroke-related phenotypes have also been identified and need further investigation.

### Methodological Considerations

This meta-analysis only collects RCTs to guarantee the reliability of its conclusions. There is no evidence of publication bias and statistical significance in the results of sensitivity analysis to ensure stability and creditability.

There exist several disadvantages. First, this meta-analysis does not exclude research that uses CSF except G-CSF, however, only one study takes EPO as an intervention, the other seven studies subcutaneously inject G-CSF, which makes subdivision and analysis impossible. If omitted, the sample size will be greatly impacted, thus decreasing the power of the results. Secondly, Sprigg (Sprigg et al., 2006) had conducted a concentration gradient of G-CSF, for fully utilizing all data the research provided, we synthesized the outcome, which means the omission of the details. Even though most trials have taken subcutaneous injection of 10 μg/kg G-CSF as an intervention, there are exceptions (Sprigg et al., 2006; Yip et al., 2011). CSF is proven to be safe on the whole, but it is not clear which kind of CSF at which maximum concentration is safe. Last but not least, in terms of quality assessment, only three out of the eight studies are ranked as having a low bias in all scales. The studies by Gorthi, Prasad, and Zhou are open-label, and thus ranked as having a high bias in the scales of “Selection bias” and “Performance bias”. The fact that patients and their families know exactly what kind of treatment they are undergoing could trigger placebo effects, which impact the real effect of CSFs. Though the study of Yip et al. (Yip et al., 2011) claims that their article is randomized, they do not describe how they randomize participants into different groups, which may also shake the reliability of our study.

### Implications for Further Studies

According to the quality assessment by GRADE. The qualities of CD34^+^, SAE (all-cause death), and SAE (recurrent stroke) are high while NIHSS and BI are low. Our analysis showed that only one study investigated the dose of G-CSF and its efficacy in humans with stroke (Sprigg et al., 2006), whereas no studies investigated CSF in stroke with other conditions such as stroke complications, CSF administration, and baseline characteristics of patients (e.g. age, smoke, alcohol abuse). Rigorously designed randomized controlled studies are needed to explore how these factors will impact the function of CSF in stroke further. Furthermore, the studies included in this meta-analysis do not investigate whether the subtypes of ischemic stroke will influence the function of CSF, while most animal studies that focus on CSF and stroke use middle cerebral artery occlusion (MCAO) models, which only mimic strokes that have happened in the middle cerebral artery territory. This may partly explain the failure of the transformation from preclinical studies to clinical studies, which should also be paid attention to in future studies.

## Conclusion

In summary, though the safety of CSF therapy has been corroborated, our meta-analysis does not provide evidence that CSF administration statistically significantly improves the neurological deficits in patients with stroke. Larger, higher-quality RCTs are necessary for fully addressing the problem, and the administration of CSFs should be undertaken with more caution.

## Data Availability

The original contributions presented in the study are included in the article/Supplementary Material, further inquiries can be directed to the corresponding authors.

## Supplementary Material

Supplementary Figure 1 Meta-regression for NIHSS.

Supplementary Figure 2 Meta-regression for Barthel index.
